# Non-Biologic Injections in Sports Medicine

**DOI:** 10.1007/s12178-026-10057-4

**Published:** 2026-09-15

**Authors:** Alan Z. Grusky, Nicolas S. Hatamiya, William A. Berrigan, Faustine D. Ramirez

**Affiliations:** 1 Department of Orthopaedic Surgery, Department of Family & Community Medicine, University of California, San Francisco, San Francisco, CA USA; 2 Department of Orthopaedic Surgery, University of California, San Francisco, 1500 Owens Street, Suite 170, San Francisco, CA 94158 USA

**Keywords:** Non-biologic injections, Sports medicine, Corticosteroid injection, NSAID injection, Ketorolac, Viscosupplementation

## Abstract

**Purpose of Review:**

Non-biologic injection therapies are among the most commonly utilized interventions in sports medicine. Yet, the evidence supporting their use varies widely across injectates and clinical indications. This review provides an overview of the most broadly used non-biologic injectates in musculoskeletal care, including corticosteroids, local anesthetics, non-steroidal anti-inflammatory drugs (NSAID), normal saline, dextrose prolotherapy, hyaluronic acid, and botulinum toxin. For each agent, we discuss the mechanism of action, adverse effect profile, and current clinical evidence across a range of sports medicine conditions.

**Recent Findings:**

Corticosteroids are the most extensively studied injectate and demonstrate consistent short-term analgesic benefit; however, data highlights dose-dependent risks to articular cartilage and tendon with implications for long-term outcomes. NSAID injections have shown comparable efficacy to corticosteroids across several indications, representing a viable alternative in appropriately selected patients. Hyaluronic acid has shown benefit in knee osteoarthritis, but guideline recommendations remain divided on its use. Dextrose prolotherapy and botulinum toxin exhibit condition-specific promise, each supported by a limited but growing evidence base. The therapeutic effects observed in saline control groups underscore the complexity of interpreting injection-based research.

**Summary:**

While several traditionally employed injectates have demonstrated efficacy for short-term pain relief and functional improvement, concerns regarding long-term tissue effects and variable clinical outcomes have prompted increasing interest in alternative therapies. Accordingly, treatment selection requires careful consideration of the underlying pathology, therapeutic goals, safety profile, and available evidence. This review synthesizes the current literature to provide clinicians with a practical, evidence-based framework for the use of non-biologic injections in sports medicine.

## Introduction

Injection therapy is a cornerstone of sports medicine care, serving both diagnostic and therapeutic roles across a wide spectrum of musculoskeletal conditions. While biologic injectates such as platelet-rich plasma have garnered significant attention in recent years, non-biologic agents—including corticosteroids, local anesthetics (LA), nonsteroidal anti-inflammatory drugs (NSAID), normal saline (NS), dextrose prolotherapy, hyaluronic acid (HA), and botulinum toxin (BT)—remain among the most widely utilized and accessible injectable therapies in clinical practice [1]. Despite their widespread use, these injectates carry distinct adverse effect profiles that must be carefully weighed against their clinical benefits. Furthermore, the quality and depth of evidence supporting each agent varies considerably across indications. This narrative review provides an overview of the most commonly used non-biologic injections in sports medicine, examining each agent’s mechanism of action, safety and side effect profile, and the current state of evidence for its clinical use in common musculoskeletal conditions.

## Corticosteroids

Corticosteroid injections (CSI) are well-studied and widely used in the treatment of both intra-articular and extra-articular musculoskeletal conditions in sports medicine [2]. 

### Mechanism of Action

Corticosteroids exert anti-inflammatory effects through genomic modulation of inflammation, inhibiting pro-inflammatory transcription factors (e.g., NF-κB, AP-1) while upregulating anti-inflammatory gene expression, thereby reducing cytokine and mediator production [3]. Their mechanism of action is also thought to include a non-genomic component via membrane stabilization and modulation of intracellular signaling, which may contribute to early analgesic effects following injection [4]. CSI are efficacious at reducing pain through these mechanisms, but these effects can be short-term, typically lasting weeks to months, with the most robust benefit occurring in the first two weeks and diminishing over time [5].

### Adverse Effects

Despite their widespread use, CSI have well-established local and systemic adverse effects and potential long-term detrimental effects. The systemic adverse effects of CSI are discussed in Table 1. Common local adverse effects from CSI include transient pain and swelling at the injection site, termed “steroid flare,” which typically occurs 24–72 h post-injection [17]. Local soft tissue reactions include fat atrophy, skin hypopigmentation, and soft tissue calcifications [5]. There is an increased risk of periprosthetic joint infection after knee, hip, and shoulder arthroplasty when a preoperative CSI is performed within three months of surgery [18, 19]. Some studies also demonstrate an increased risk of post-surgical infection after arthroscopy following a preoperative CSI [20]. Septic arthritis is a rare but serious complication, with incidence reported between 0.03 and 0.08% [21–23].

**Table 1:** Systemic Adverse Effects of Non-Biologic Injection Agents

Agent	Systemic Adverse Effects
Corticosteroids	Systemic adverse effects of corticosteroids include transient hyperglycemia, transient hypertension, hypersensitivity reactions, facial flushing, decreased bone mineral density (with cumulative exposure), adrenal suppression, progression of glaucoma and retinopathy, insomnia, mood changes, and irregular menstruation [5–7]. These systemic effects are primarily self-limited, though may warrant counseling for high-risk populations including premenopausal women, patients with diabetes and/or hypertension, and pediatric patients.
Local Anesthetics	Systemic adverse effects of local anesthetics are rare and typically occur only with inadvertent intravascular or intrathecal administration; these include serious central nervous system (CNS) and cardiovascular events such as seizures, CNS depression, arrhythmias, and cardiac arrest [8].
Non-Steroidal Anti-Inflammatory Drugs	Systemic adverse effects of NSAIDs include gastrointestinal effects (e.g. ulcers, perforation, and bleeding), cardiovascular thrombosis, renal impairment, hepatotoxicity, and increased bleeding risk [9, 10]. Despite lower systemic exposure with intra-articular or peritendinous injection, special considerations should still be made for patients deemed high-risk or contraindicated for NSAID therapy, including those with active peptic ulcer disease, history of gastrointestinal bleeding, advanced renal impairment, high bleeding risk, aspirin-exacerbated respiratory disease, current pregnancy, and concomitant use of other NSAIDs, anticoagulants, angiotensin converting enzyme (ACE) inhibitors, angiotensin II receptor blockers (ARBs), and diuretics. Additionally, caution should be taken in older patients and those with cardiovascular disease given the class-wide risk of cardiovascular thrombotic events associated with NSAIDs [11–13].
Normal Saline	No serious systemic adverse effects have been reported across clinical trials.
Dextrose Prolotherapy	No serious systemic adverse effects have been reported across clinical trials.
Hyaluronic Acid	Most studies do not report any serious systemic adverse effects for hyaluronic acid (HA). However, one meta-analysis found a statistically significant increased risk of serious adverse events (defined as events resulting in hospital admission, prolongation of hospital stay, persistent or major disability, congenital abnormality of offspring, life threatening events, or death) with HA compared to placebo [14].
Botulinum Toxin	All commercial botulinum toxin (BT) products carry a warning regarding the potential for spread of the toxin beyond the injection site, which may produce serious adverse effects including dysphagia, dysphonia, generalized weakness, and respiratory compromise [15]. However, these systemic effects are rare, especially at doses typically used in sports medicine (20-100U), and appear to correlate more with total injection dose, injection site, and re-injection frequency [16].

Basic science and animal studies demonstrate corticosteroid chondrotoxicity through catabolic effects on cartilage proteins (aggrecan, type II collagen, proteoglycans), reduced chondrocyte viability, and altered cartilage morphology [24–27]. In vitro studies suggest that chondrotoxicity may vary among corticosteroid preparations, with betamethasone associated with the greatest toxicity, methylprednisolone and triamcinolone demonstrating intermediate effects, and dexamethasone exhibiting the least toxicity toward chondrocytes and mesenchymal stem cells [28–30]. Clinical evidence also suggests detrimental effects of CSI on cartilage health both in patients with and without pre-existing degenerative joint disease, including a randomized control trial which demonstrated that repeated intra-articular triamcinolone resulted in significantly greater cartilage volume loss in the knee compared to saline injections [31, 32]. Large prospective clinical studies have found that CSI in patients with hip pain and knee pain without pre-existing osteoarthritis were associated with a dose-dependent increased risk of total hip and knee arthroplasty respectively at 5 years, with each additional CSI conferring increased risk for arthroplasty [32–34]. However, a recent study using physiologically relevant concentrations of triamcinolone on intact healthy and osteoarthritic human cartilage found no measurable chondrocyte toxicity and only modest, reversible suppression of matrix synthesis, suggesting prior in vitro models may overestimate clinical risk [35].

CSI also appear to have adverse effects on tendon, with in vitro studies suggesting decreased tendon cell viability, proliferation, architecture, collagen synthesis, collagen organization, and mechanical properties [36–38]. Piper et al. found that while dexamethasone alone was not significantly toxic to tenocytes after exposure, it potentiated the toxicity of ropivacaine at higher doses [39]. Clinically, CSI are associated with worse long-term outcomes in the treatment of tendinopathies, with most of this evidence seen in lateral epicondylopathy [40, 41]. One meta-analysis showed that CSI was the most consistent risk factor for revision surgery after arthroscopic rotator cuff repair, with patients receiving CSI having a 47% increased risk of revision surgery [42]. The risk of tendon rupture after corticosteroid injection is well-described but appears relatively low, although incidence data vary widely in the literature (0.1–10%) [41, 43].

Finally, intra-articular CSI have also been associated with adverse osseous effects including subchondral insufficiency fracture (0.3%), avascular necrosis (0.6–20.4%), and rapidly destructive hip disease with bone loss (3–7%) [44–46].

### Clinical Indications

Clinical indications for corticosteroid injections are broad, including osteoarthritis, inflammatory arthropathies, adhesive capsulitis, subacromial impingement, tendinopathies and tenosynovitis, and bursitis, among many others [5]. A review of the clinical evidence supporting the use of CSI for each individual indication is beyond the scope of this article.

## Local Anesthetics

Local anesthetics (LA) are commonly used in sports medicine for multiple indications, including procedural local and regional anesthesia (peripheral nerve blocks) along with diagnostic and therapeutic injections. The most commonly used LAs in sports medicine are lidocaine, bupivacaine, and ropivacaine [47]. Among these, lidocaine has the fastest onset of action and shortest duration of effect, while bupivacaine has the slowest onset and longest duration of effect, and ropivacaine is intermediate but has a profile closer to that of bupivacaine [48].

### Mechanism of Action

Local anesthetics work by reversibly inhibiting voltage-gated sodium channels and preventing initiation and propagation of action potentials, thereby blocking nociceptive signal transmission [49, 50]. These effects are use- and state-dependent, with variability in onset and duration based on lipid solubility, protein binding, and tissue pharmacokinetics of each individual anesthetic [48, 51].

### Adverse Effects

The systemic adverse effects of LAs are discussed in Table 1. With regards to local adverse effects, the chondrotoxic effects of LAs on articular cartilage have been well-established in the literature [25, 52–61]. A recent review summarized this literature in human knee articular cartilage, showing a dose-dependent and time-dependent chondrotoxicity across all LAs studied, which may be exacerbated by concurrent corticosteroid use [62]. Ropivacaine at concentrations below 0.75% appeared to be the least toxic, while bupivacaine appears to be the most chondrotoxic, particularly at concentrations of 0.5% and higher [62].

LAs also exert detrimental effects on tendon and muscle tissue. One study found a dose-dependent toxicity of 1% and 2% lidocaine on bovine tenocytes, but did not see this same effect with ropivacaine or dexamethasone alone [39]. A separate study culturing human rotator cuff tenofibroblasts with ropivacaine, bupivacaine, and lidocaine at multiple concentrations confirmed a concentration- and time-dependent cytotoxicity across all agents, with lidocaine demonstrating high toxicity even at very low concentrations, and 0.2% ropivacaine demonstrating the least toxicity [63], consistent with the previously cited study [55]. Regarding myotoxicity, evidence suggests that bupivacaine causes greater muscle necrosis than ropivacaine [64].

There is also evidence of time- and concentration-dependent neurotoxicity of LAs to peripheral nerve tissue. In vitro and animal studies have shown effects in Schwann cells, axonal degeneration, and demyelination, with bupivacaine demonstrating greater toxicity than lidocaine or ropivacaine at equivalent concentrations [65–68]. Clinically, the incidence of neurologic symptoms (e.g. paresthesia, prolonged numbness, dysesthesia, motor weakness) following the injection of LAs around peripheral nerves is relatively low (0.02–2.2%), however intrafascicular injection increases this risk [69, 70].

### Clinical Indications

From a clinical perspective, LAs are frequently used in peripheral nerve blocks (with or without adjuvant agents such as corticosteroids) around specific peripheral nerves to interrupt nociceptive transmission from a defined anatomic region [71, 72]. They are used increasingly in sports medicine for perioperative or periprocedural pain management as well as for diagnostic and/or therapeutic interventions for musculoskeletal pain conditions [73]. The introduction of ultrasound guidance has improved the overall success, accuracy, and efficacy of peripheral nerve blocks [74, 75], with complication rates in one study shown to be between 0 and 4.8% [72]. The most common complications include muscular weakness, falls, neuropathy, intravascular/intraneural injection, and hematoma. Neurologic symptoms are predominantly sensory and transient, with 73% resolving within the first week and long-term deficits estimated at 2–4 per 10,000 patients [76, 77]. Rebound pain—an abrupt increase in pain severity after block resolution—occurs in up to 50% of patients [78, 79]. A narrative review found that lower extremity nerve blocks consistently reduced postoperative pain scores, opioid use, and nausea following hip and knee arthroscopy [72]. A meta-analysis evaluating genicular nerve blocks for knee osteoarthritis demonstrated pain reduction at one and three months that met the minimal clinically important difference (MCID) [80]. There is also a large body of evidence supporting the use of suprascapular nerve blocks for multiple etiologies of chronic shoulder pain, including glenohumeral osteoarthritis, adhesive capsulitis, and rotator cuff disease [73, 81–84].

## Non-Steroidal Anti-Inflammatory Drugs

NSAIDs have long been used in the treatment of musculoskeletal conditions to reduce pain and inflammation. Traditionally, NSAIDs have been FDA-approved and commonly used via oral, topical, and intramuscular routes of administration. However, within the last decade, more recent clinical studies have evaluated the off-label use of intra-articular or peritendinous NSAID injections to achieve targeted local analgesic and anti-inflammatory effects with few systemic side effects, theoretically conferring a more favorable safety profile compared to long-term oral use.

Pharmacokinetic studies have demonstrated that a single intra-articular NSAID injection leads to a two- to three-fold higher synovial maximum NSAID concentration compared to an equivalent oral dose [11]; however, the medication quickly diffuses out of the joint space into the plasma with a synovial fluid half-life ranging from 1.5 to 7 h [85]. Despite rapid synovial clearance, the prolonged clinical efficacy likely reflects sustained cyclooxygenase inhibition and peripheral nociceptor desensitization from the initial peak drug concentration [11, 86]. Overall, the systemic absorption from a single intra-articular NSAID injection is approximately ten-fold less than a one-week course of oral NSAIDs and comparable to a one-week course of topical NSAIDs [11, 87]. Ketorolac is the most widely studied and available NSAID in the United States for intra-articular or peritendinous use. It is available in injectable solutions of 15 mg/mL and 30 mg/mL, with most clinical studies using a dose of 30 to 60 mg.

### Mechanism of Action

NSAIDs exert their effects through a mechanism of action that inhibits cyclooxygenase enzymes, blocking the conversion of arachidonic acid to prostaglandins and other inflammatory mediators [88].

### Adverse Effects

Systemic adverse effects of NSAIDs are discussed in Table 1. With regards to local adverse effects, several studies have evaluated NSAIDs and their potential adverse effects on articular cartilage and tendons, with mixed results [89]. One in vitro study exposing human articular cartilage to single-dose equivalents of ketorolac found a dose-dependent cytotoxic effect on chondrocytes—however, a constant medication delivery system was used which may have overestimated toxicity relative to a single clinical injection [90]. Other studies have not found evidence of chondrotoxicity in human chondrocytes after exposure to NSAIDs, demonstrating no decrease in chondrocyte viability [91, 92], no longitudinal changes in cartilage thickness, kinematics, or histology [93], and no differences in chondrocyte death, apoptosis-related gene expression, or anabolic gene expression [94]. In regards to tendon health, there is preclinical evidence linking NSAIDs to tenotoxicity [95–98], but there is limited data examining ketorolac specifically. In a rabbit model, multiple sequential peritendinous ketorolac injections caused no significant histologic or biomechanical tendon damage compared to saline and corticosteroids [99]. While one study showed an increase in tenocyte proliferation after human tenocytes were treated with ketorolac [91], another in vitro study examining human supraspinatus tenocytes found a significant reduction in real-time cell index at both 15 and 30 mg concentrations of ketorolac, suggesting tenocyte cytotoxicity [100]. In summary, current evidence suggests that ketorolac is likely safe for articular cartilage but data are more conflicting for tendon, indicating possible dose-dependent tenotoxicity; more research is warranted to further elucidate these potential effects and long-term safety.

### Clinical Indications

Ketorolac injections have been well-studied for several clinical indications. A systematic review examined multiple comparative studies of NSAID versus CSI for upper and lower extremity musculoskeletal conditions [101]. Subacromial impingement syndrome was the most thoroughly studied pathology in this review; several meta-analyses have shown that both NSAIDs and CSI produced significant improvements in shoulder pain and function, with no significant differences in pain scores at one- and three-month follow-up, no meaningful differences in adverse event rates, and a favorable cost profile for NSAIDs [101, 102]. In adhesive capsulitis, evidence for intraarticular NSAID injections is limited and does not currently support its use [103–105]. In the lower extremity, there is clinical data supporting the use of intra-articular NSAIDs in both knee and hip joints. In the aforementioned systematic review, both NSAIDs and CSI significantly improved pain for knee osteoarthritis in all but one study, with no significant differences between groups at one or three months in the meta-analysis [101]. Functional outcomes measured by WOMAC showed improvement in both groups, though two studies favored CSI [101]. In hip osteoarthritis, a retrospective study found that ultrasound-guided intra-articular ketorolac was as effective as triamcinolone, with no significant differences in Harris hip scores, pain scales, or success rates at one, three, and six months [106]. A recent retrospective matched cohort study found no significant differences between NSAID and CSI in pain relief, duration of effect, time to effect onset, or adverse effects among hip injections for both intra-articular (hip osteoarthritis, femoroacetabular impingement syndrome, labral tear) and extra-articular pathology (gluteal or iliopsoas tendinopathy) [107].

## Normal Saline

NS injections have traditionally served as placebo control arms in injection trials, but a large body of evidence demonstrates that these injections seem to confer clinically and statistically meaningful therapeutic effects.

### Knee Osteoarthritis

In knee osteoarthritis, a recent review examining NS placebo effect sizes in randomized controlled trials (RCTs) found that 93% of placebo data points demonstrated pain reduction from baseline, of which nearly half exceeded the MCID. Similarly, a meta-analysis examining intra-articular NS injections as comparator arms across trials of knee osteoarthritis found that NS injections significantly improved short-term knee pain (three months or less) in 32 studies and long-term knee pain (six to twelve months) in 19 studies [108]. Another meta-analysis of patients with knee osteoarthritis found statistically and clinically significant improvements in pain and function after intra-articular NS injections [109].

### Tendinopathy

The therapeutic effect of NS injections is not limited to joints and may also confer benefit for tendon pathology. A recent systematic review found significant reductions in pain and function up to 12 months after NS injection among patients with plantar fasciitis, exceeding the established MCID; notably, no significant change in plantar fascia thickness was observed, suggesting the therapeutic effect may operate primarily through neurochemical or pain-sensitization pathways rather than structural remodeling [110]. Similar findings have been reported in lateral epicondylitis [111, 112]. One systematic review found statistically and clinically significant reduction in pain and function after NS injection, with subgroup analysis comparing NS injection to no intervention demonstrating consistent results [111]. A systematic review examined 14 placebo-controlled RCTs of patellar tendinopathy interventions, and found that injected NS placebo was more effective than non-injected placebo modalities, suggesting that efficacy of injection-based therapies may be partly attributable to an enhanced placebo component from injection rather than true therapeutic superiority [113].

### Mechanism of Action

Multiple hypotheses have been proposed to explain the effects of saline placebo injections, including patient-related factors (baseline pain severity, prior treatment experience, expectations), trial design factors, provider behavior (clinician confidence and optimism), and biological mechanisms (aspiration prior to injection, dilution of intra-articular inflammatory mediators, changes in joint osmolality, and localized cellular responses to needling and/or hydrodissection) [110, 114, 115]. The relative contributions of each of these factors remain incompletely understood and warrant further investigation. However, it is clear that NS placebo injection arms in clinical trials consistently demonstrate meaningful therapeutic benefit—recognition of this is crucial for interpretation of research on injection treatments in sports medicine and should be carefully considered in the design of future clinical trials.

### Clinical Indications

In clinical practice, NS is commonly used in nerve hydrodissection procedures, in which an injection of fluid around a peripheral nerve is used to mechanically separate the nerve from surrounding tissue. Hydrodissection is thought to exert a therapeutic effect through mechanical decompression of the nervi nervorum and vasa nervorum at the epineurium, mechanical disruption of surrounding perineural adhesions and fibrosis, and reduction of nerve gliding resistance [116–118]. This may be performed with NS, corticosteroids, dextrose, platelet-rich plasma (PRP), or other injectates [119]. Multiple trials studying carpal tunnel syndrome have shown patient improvement after hydrodissection of the median nerve with exclusively or majority NS [120, 121]. Data remain very limited for hydrodissection of other peripheral nerve entrapment syndromes beyond carpal tunnel syndrome [117, 119, 122]. Beyond small-volume NS perineural injections, high-volume distension or “hydrodilatation”—the injection of large volumes of fluid which typically includes NS with or without LA and/or corticosteroid—has demonstrated therapeutic benefit in adhesive capsulitis. This treatment appears to provide mild but meaningful short-to-medium-term improvements in pain, function, and range of motion compared to other conservative treatments such as intra-articular CSI alone or physical therapy alone [123, 123, 124].

## Dextrose Prolotherapy

Prolotherapy is an injection technique that introduces a non-biologic irritant solution—most commonly hyperosmolar dextrose—to sites of musculoskeletal pathology. In clinical practice, hypertonic dextrose solutions are often diluted with LA (with a total volume generally ranging from 5 to 10 mL) before being delivered into the target tissue or joint. Most studies evaluating prolotherapy have used dextrose concentrations ranging from 10% to 25%, with higher concentrations typically used in intraarticular injections and a broader range of concentrations employed for tendons. Treatment protocols often involve multiple sessions—typically three to five injections administered at three- to six-week intervals, though considerable heterogeneity in dosing, concentration, and frequency exists across the published literature [125, 126].

### Mechanism of Action

It is thought that at higher concentrations of 12.5% or greater, dextrose prolotherapy induces localized cellular stress that triggers an inflammatory-proliferative cascade, thereby upregulating proinflammatory mediators, recruiting growth factors, and stimulating connective tissue repair [127–129]. Another proposed theory independent of this inflammatory pathway suggests that dextrose exerts a direct neurogenic analgesic effect by activating ASIC1a channels on muscle, triggering release of substance P and causes downstream signaling that reduces chronic mechanical hyperalgesia [130].

### Adverse Effects

Dextrose prolotherapy has a favorable clinical safety profile with no serious or systemic adverse events reported across multiple systematic reviews. The most common local adverse effects are transient injection-site pain and ecchymosis, both of which are self-limited [131–133]. Furthermore, existing pre-clinical evidence suggests that dextrose is neither chondrotoxic nor tenotoxic. In fact, hyperosmolarity may even be chondroprotective with studies demonstrating that increased osmolarity reduces chondrocyte death in injured cartilage compared to isotonic conditions [134, 135] and other studies suggesting hypertonic dextrose enhances cartilage matrix synthesis [136]. In an in vivo study using a rat Achilles tendon model, 12.5% dextrose was not found to be deleterious to tendon tissue, as there was no change in maximum load at failure, absorbed energy, or histological properties when compared to controls [137].

### Clinical Indications

Among sports medicine conditions, the strongest clinical evidence for dextrose prolotherapy is found in lateral epicondylopathy. A meta-analysis of eight RCTs found that dextrose prolotherapy was superior to a variety of active controls at 12 weeks for both pain and function [133]. A recent RCT with two-year follow-up found that both PRP and prolotherapy yielded better clinical and functional outcomes than ESWT and physiotherapy in chronic lateral epicondylosis, with prolotherapy showing significantly greater improvement on DASH scores than physiotherapy at 18 months [127]. Current evidence also supports dextrose prolotherapy in the treatment of knee osteoarthritis. A meta-analysis found that dextrose prolotherapy had favorable effects on pain, function, and quality of life compared to placebo and noninvasive controls, with dose-dependent and time-dependent effects [128]. A separate systematic review concluded that dextrose prolotherapy may offer benefits for pain reduction and functional improvement in osteoarthritis, but cautioned that the evidence is limited by a high risk of bias across studies [129]. Data are more limited for other sports medicine conditions. In plantar fasciitis, systematic reviews have found dextrose prolotherapy to be inferior to CSI in the short term, but superior in the medium- and long-term for sustained pain relief [130, 138, 139]. For rotator cuff pathology, studies suggest dextrose prolotherapy yields better long-term outcomes compared to CSI [140] and NS [141], and may be an effective adjuvant to physical therapy, although there is a significant variation among protocols in these studies [131]. There is limited or mixed evidence for dextrose prolotherapy in the treatment of sacroiliac joint pain [142] and Achilles tendinopathy [143].

## Hyaluronic Acid

HA is a high-molecular-weight glycosaminoglycan that is a natural component of synovial fluid, cartilage, and the extracellular matrix of soft tissues. In sports medicine, exogenous HA is typically administered via intra-articular or peritendinous injection, a practice broadly termed “viscosupplementation” [14, 144].

Commercially available HA preparations vary considerably in molecular weight, degree of cross-linking, source, and dosing regimen, ranging from a single injection to a course of three to five weekly injections [145]. The effect of molecular weight on the clinical efficacy of intra-articular hyaluronic acid remains a contested topic, as some evidence favors higher molecular weight formulations [145, 146] while other studies and clinical guidelines do not support this [147, 148]. The current evidence does not definitively establish the superiority of one molecular weight category over another across all patient populations.

### Mechanism of Action

The proposed mechanism of action of hyaluronic acid (HA) is multifactorial including chondroprotection, stimulation of proteoglycan and glycosaminoglycan synthesis, anti-inflammatory activity, and direct analgesic effects [149]. At the molecular level, there is evidence that HA downregulates pro-inflammatory cytokines, inhibits matrix metalloproteinases that degrade cartilage, scavenges reactive oxygen species, and modulates nociceptive pathways [150]. There may also be a mechanical lubricant effect, as exogenous HA appears to stimulate endogenous HA production [151, 152].

### Adverse Effects

Systemic adverse effects of HA are discussed in Table 1. The most common local adverse effects after HA injection are transient local reactions (e.g. injection site pain, swelling, arthralgia), and the incidence of these reactions is similar to intra-articular placebo injections [153]. Pseudoseptic arthritis (an acute inflammatory flare that mimics joint infection) has a reported incidence ranging from 0 to 5.6% per injection across studies [154, 155], and appears to be disproportionately observed in the high-molecular-weight, cross-linked hylan formulation (Synvisc) [156].

### Clinical Indications

Knee osteoarthritis is the most extensively studied clinical use for hyaluronic acid injections. A large meta-analysis of 169 trials found that viscosupplementation led to a statistically significant reduction in pain compared to placebo, which did not exceed the MCID [14]. A more recent review suggested that most existing studies found statistically significant benefits of HA on pain and function in knee osteoarthritis, attributing negative conclusions to restrictive inclusion criteria [157]. Patients who are younger (< 65 years old), with higher BMI (≥ 25), more severe baseline symptoms, and less severe disease (Kellgren-Lawrence grade ≤ 2) appear to derive more benefit from HA [158, 159]. Clinical guidelines surrounding the use of HA for knee osteoarthritis are discordant—the American College of Rheumatology [160] and American Academy of Orthopaedic Surgeons [147] conditionally recommend against routine use of HA, while it is recommended as a second-line treatment by the Osteoarthritis Research Society International [161] and the European Society for Clinical and Economic Aspects of Osteoporosis, Osteoarthritis and Musculoskeletal Diseases [162]— highlighting the heterogeneity of expert opinion surrounding its use for knee osteoarthritis. HA has also been studied in hip, shoulder, hand, and ankle osteoarthritis to varying degrees, with most studies demonstrating little or no benefit over control treatments [14, 163–165].

HA is less commonly used to treat soft tissue conditions in sports medicine. However, a recent meta-analysis evaluated HA across a wide range of soft tissue pathology, including rotator cuff disease, lateral epicondylitis, ankle sprains, achilles tendinopathy, and patellar tendinopathy. Pooled results found statistically significant pain reduction over controls at both 6 weeks and greater than 12 weeks [166], suggesting potential efficacy in soft tissue pathology. An RCT comparing subacromial HA injections with physical therapy for shoulder tendinopathy found HA superior in pain control, disability reduction, and range of motion, with benefits lasting at least three to six months [167]. Although current evidence remains limited, these findings suggest that HA may have potential benefit and warrant further investigation.

## Botulinum Toxin

BT is a neurotoxic protein produced by the bacterium *Clostridium botulinum*. A total of seven serotypes have been isolated, with serotypes A and B used clinically [168, 169]. BT is currently FDA-approved for cervical dystonia, spasticity, and chronic migraine; however, it has gained attention for off-label use in a variety of sports medicine conditions.

### Mechanism of Action

Botulinum toxin (BT) reversibly inhibits acetylcholine release at the neuromuscular junction by cleaving SNARE proteins, causing temporary chemodenervation and muscle relaxation [170, 171]. In addition, BT also exerts direct analgesic effects by inhibiting the release of pain-mediating neuropeptides such as substance P, CGRP, and glutamate, thereby reducing peripheral sensitization [172]. These mechanisms allow for motor relaxation and nociceptive modulation, with effects typically lasting between three and six months before nerve terminal regeneration restores to baseline [173].

### Adverse Effects

Systemic adverse effects of BT are discussed in Table 1. Most local adverse effects associated with BT injections are mild and self-limited, including injection site pain, irritation, and transient muscle weakness. Meta-analyses of BT for plantar fasciitis and intra-articular use found no significant difference in adverse event rates between BT and control groups [174, 175]. Dosing and injection protocols vary considerably across the published literature, and this heterogeneity remains a significant limitation of the available evidence base.

### Clinical Indications

Among sports medicine clinical applications, plantar fasciitis has the most robust evidence supporting the use of BT. One meta-analysis found that BT provided significant pain relief over placebo at both short-term and six-month follow-up [176]. A more recent meta-analysis of seven RCTs demonstrated statistically significant pain reduction and sustained functional improvements for up to one year after BT injection compared to controls [174]. Evidence also suggests benefit in the treatment of lateral epicondylopathy. An RCT evaluating treatment-resistant lateral epicondylopathy found statistically significant pain reduction in the BT group compared to NS controls [177]. A recent meta-analysis showed that BT significantly reduced pain at two to twelve weeks compared to placebo, although the overall study size was low [178]. BT is also used to treat chronic exertional compartment syndrome (CECS), although evidence is limited. A foundational case series found that intramuscular compartment pressures at one minute post-exercise fell by 63% in the anterior compartment and 68% in the lateral compartment after injection of BT, with associated elimination of exertional pain in 94% of patients [179]. A small retrospective study of both upper- and lower-limb CECS using BT as first-line therapy showed an initial efficacy of 68.75%, although many patient had recurrence of symptoms after several months [180]. BT is also used to treat neurogenic thoracic outlet syndrome—injection into the scalene and pectoralis minor muscles have been shown to provide short-term symptom relief in observational data, though the only RCT showed no significant benefit over placebo [181–183]. Finally, intraarticular BT has demonstrated benefit for knee osteoarthritis in multiple meta-analyses, with short-term pain reduction compared to placebo and similar efficacy to CSI [184, 185]. However, evidence for durable functional improvement remains limited, and more investigation is needed on the role of BT in sports medicine.

## Conclusions

Non-biologic injections remain a fundamental component of non-operative care in sports medicine, offering clinicians a diverse set of tools to treat pain and functional limitation. However, the breadth and depth of evidence varies considerably across agents and indications, and high-quality clinical trials remain limited for many of these therapies. As such, clinicians should consider non-biologic injections within a comprehensive, multimodal management strategy and adopt an individualized, evidence-based approach to treatment selection that balances efficacy, safety, and patient-specific goals.

## Key References

- Benzon HT, Provenzano DA, Nagpal A, Souza D, Eckmann MS, Nelson AM, et al. Use and safety of corticosteroid injections in joints and musculoskeletal soft tissue: guidelines from the American Society of Regional Anesthesia and Pain Medicine, the American Academy of Pain Medicine, the American Society of Interventional Pain Physicians, and the International Pain and Spine Intervention Society. Reg Anesth Pain Med. 2026;51:746–73. https://doi.org/10.1136/rapm-2024–105656
  - ○A comprehensive multi-society guideline on corticosteroid injection use and safety in peripheral joints and musculoskeletal soft tissues. Provides evidence-graded recommendations on efficacy, dosing, frequency, and adverse effects of corticosteroid injections.
- Fackler NP, Yareli-Salinas E, Callan KT, Athanasiou KA, Wang D. In Vitro Effects of Triamcinolone and Methylprednisolone on the Viability and Mechanics of Native Articular Cartilage. Am J Sports Med. 2023;51:2465–71. https://doi.org/10.1177/03635465231162644
  - ○A study demonstrating that a single clinically relevant corticosteroid exposure significantly reduces the mechanical properties of native articular cartilage. Provides a mechanistic basis for dose-dependent chondrotoxicity concerns raised in clinical studies.
- Selig DJ, Kress AT, Horton IM, Livezey JR, Sadik EJ, DeLuca JP. Pharmacokinetics, safety and efficacy of intra-articular non-steroidal anti-inflammatory drug injections for the treatment of osteoarthritis: A narrative review. Journal of Clinical Pharmacy and Therapeutics. 2022;47:1122–33. https://doi.org/10.1111/jcpt.13669
  - ○A comprehensive review of intra-articular NSAID pharmacokinetics, safety, and efficacy. Synthesizes preclinical and clinical data on local versus systemic drug exposure, adverse effect profiles, and clinical outcomes from randomized controlled trials across NSAID preparations.
- Rhim HC, Ruiz J, Taseh A, Afunugo W, Crockett Z, Schon J, et al. Nonsteroidal Anti-Inflammatory Drug Injections versus Steroid Injections in the Management of Upper and Lower Extremity Orthopedic Conditions: A Systematic Review with Meta-Analysis. J Clin Med. 2024;13:1132. https://doi.org/10.3390/jcm13041132
  - ○A large systematic review and meta-analysis directly comparing injectable NSAIDs to corticosteroids across multiple musculoskeletal conditions. Supports NSAID injections as a safe and effective alternative to corticosteroid injections.
- Acosta-Olivo CA, Millán-Alanís JM, Simental-Mendía LE, Álvarez-Villalobos N, Vilchez-Cavazos F, Peña-Martínez VM, et al. Effect of Normal Saline Injections on Lateral Epicondylitis Symptoms: A Systematic Review and Meta-analysis of Randomized Clinical Trials. Am J Sports Med. 2020;48:3094–102. https://doi.org/10.1177/0363546519899644
  - ○A systematic review and meta-analysis demonstrating significant improvements in pain and function for lateral epicondylitis with normal saline injections. Reinforces the complexity of interpreting injection-based clinical research, suggesting that needle insertion and/or volume distension may independently contribute to therapeutic effect.
- Bruyère O, Alokail M, Al-Daghri N, Reginster J-Y, Sabico S. Effects of intra-articular hyaluronic acid injections on pain and function in patients with knee osteoarthritis: An umbrella review of systematic reviews and meta-analyses of randomized placebo-controlled trials. Maturitas. 2025;203:108779. https://doi.org/10.1016/j.maturitas.2025.108779
  - ○ A high-level umbrella review on hyaluronic acid for knee osteoarthritis demonstrating that higher quality systematic reviews reported benefits on pain and function. Suggests that negative conclusions in the literature are driven by restrictive inclusion criteria and debates on clinical relevance thresholds, rather than absence of therapeutic effect.
- Cao Y, Cai R, Han S, Li Z, Ma K, Zhou Z, et al. Quantitative analysis of the efficacy and associated factors of intra-articular hyaluronic acid with respect to osteoarthritis symptoms: A systematic review of randomized trials and model-based meta-analysis. Osteoarthritis Cartilage. 2025;33:666–79. https://doi.org/10.1016/j.joca.2025.01.008
  - ○A systematic review and meta-analysis of hyaluronic acid for knee osteoarthritis uniquely characterizing the temporal trajectory of efficacy and identifying patient subgroups most likely to benefit. Demonstrates greater benefit of hyaluronic acid in patients aged < 65 years, with BMI ≥ 25, and with Kellgren-Lawrence grade ≤ 2, providing a framework for individualized patient selection.
- Chen Y-W, Lin Y-N, Chen H-C, Liou T-H, Liao C-D, Huang S-W. Effectiveness, Compliance, and Safety of Dextrose Prolotherapy for Knee Osteoarthritis: A Meta-Analysis and Metaregression of Randomized Controlled Trials. Clin Rehabil. 2022;36:740–52. https://doi.org/10.1177/02692155221086213
  - ○ A meta-analysis of dextrose prolotherapy for knee osteoarthritis demonstrating favorable effects on pain, function, and quality of life. Suggests that combined intra-articular and extra-articular injection techniques may produce stronger effects.
- Fong HPY, Zhu M-T, Rabago DP, Reeves KD, Chung VCH, Sit RWS. Effectiveness of Hypertonic Dextrose Injection (Prolotherapy) in Plantar Fasciopathy: A Systematic Review and Meta-analysis of Randomized Controlled Trials. Arch Phys Med Rehabil. 2023;104:1941-1953.e9. https://doi.org/10.1016/j.apmr.2023.03.027
  - ○ A systematic review and meta-analysis of prolotherapy for plantar fasciopathy, finding prolotherapy superior to saline for medium-term outcomes but inferior to corticosteroids for short-term outcomes. Helps define the role of prolotherapy relative to established treatments.
- Li Q, Zhang J, Sun J, Gao C, Zhao J. Therapeutic efficacy and safety of botulinum toxin A injection in plantar fasciitis: A systematic review and meta-analysis. PLoS One. 2024;19:e0312908. https://doi.org/10.1371/journal.pone.0312908
  - ○ A systematic review and meta-analysis of botulinum toxin A for plantar fasciitis, demonstrating significant pain reduction at one month and sustained functional improvement through twelve months. Supports botulinum toxin as an effective treatment, particularly for patients who have failed or have contraindications to corticosteroid injection.

## Data Availability

No datasets were generated or analysed during the current study.
